# *Salmonella* Stanley ST29 carrying IncHI2/ST3-*bla*_NDM-5_ plasmid emerged in a 4-month-old infant with diarrhea

**DOI:** 10.1128/aac.01471-25

**Published:** 2026-02-04

**Authors:** Yu-Man Bai, Xiao-Juan Gao, Chao Yue, Zhong-Peng Cai, Guo-Long Gao, Lu-Chao Lv, Yi-Hua Cai, Hong-Mei Mo, Jian-Hua Liu

**Affiliations:** 1 State Key Laboratory for Animal Disease Control and Prevention, Key Laboratory of Zoonosis of Ministry of Agricultural and Rural Affairs, College of Veterinary Medicine, South China Agricultural University 12526, Guangzhou, China; 2 Medical Laboratory of Shenzhen, Luohu Hospital Group Luohu People’s Hospital, Shenzhen, China; University of Fribourg, Fribourg, Switzerland

**Keywords:** *Salmonella*, carbapenem-resistant *Enterobacterales *(CRE), infant, plasmid

## Abstract

*Salmonella* serovar Stanley ST29 is an emerging foodborne pathogen with community spread potential, while its carbapenem resistance remains uncommon. In a longitudinal pediatric case with diarrhea, two clonal ST29 isolates sampled 2 months apart revealed within-host evolutionary dynamics: Tn*7051*-mediated excision of *bla*_NDM-5_ from an IncHI2/ST3 plasmid. Moreover, phylogenomics connected the case isolate to strains from a healthy carrier and from food-chain-associated sources in the same region (17–18 SNPs), underscoring community dissemination and the need for One-Health surveillance.

## INTRODUCTION

*Salmonella* is a major foodborne pathogen of global concern, causing an estimated 93.8 million gastroenteritis cases and over 150,000 deaths annually, with infants and the elderly particularly vulnerable (1, 2). Antimicrobial therapy remains the cornerstone of treatment, yet resistance is rising, and carbapenem-resistant *Salmonella* has shown an increasing trend in recent years (3, 4). Within the broader emergence of carbapenem-resistant *Salmonella*, *S*. Stanley has become increasingly noteworthy (5, 6), with sporadic reports of carbapenemase genes since the first identification of *bla*_NDM-1_ (7), raising clinical concern despite the limited number of cases.

On 9 October 2024, a 4-month-old infant was admitted with diarrhea lasting 8 days, characterized by ≥5 watery stools per day and a single febrile episode at onset. The patient was diagnosed with diarrhea and intestinal dysbiosis and received probiotics with supportive care. On 14 December, the infant was re-admitted for persistent diarrhea and nasopharyngitis. Laboratory tests showed a reduced neutrophil ratio (33.7%, normal: 50%–70%), elevated lymphocyte ratio (53.7%, normal: 20%–40%), and elevated monocyte ratio (8.9%, normal: 1%–8%). Fecal examination showed watery, yellowish-brown stools. Treatment included a 3-day course of oral antibiotics, montmorillonite, and nasal irrigation. During history taking, caregivers reported that feeding bottles were not always promptly cleaned or disinfected, suggesting potential household hygiene lapses. Fecal samples were collected for sentinel testing.

Two strains of *S*. Stanley ST29, designated GD24LH212S and GD24LH266S, respectively, were isolated from fecal samples of the infant patient on 9 October and 14 December 2024, respectively. Antimicrobial susceptibility testing revealed that GD24LH212S was resistant to cephalosporins (ceftazidime, ceftriaxone, cefoperazone/sulbactam, cefepime), β-lactam/β-lactamase inhibitor combinations (amoxicillin-clavulanate, piperacillin-tazobactam), and carbapenems (ertapenem, imipenem), while GD24LH266S exhibited restored susceptibility to cephalosporins and carbapenems, but retained resistance to trimethoprim-sulfamethoxazole and penicillin (Table 1). To investigate the clonal relationship of these two isolates, we generated complete genomic data using the 2 × 150 PE Illumina HiSeq platform (200× coverage) (Illumina, San Diego, CA, USA) and QitanTech nanopore platform (QitanTech, China) and assembled the genomes with Unicycler v0.4.7 (8). Both isolates were identified as the emerging *S*. Stanley ST29 lineage, carrying highly similar plasmids and resistance genes. GD24LH212S carried IncHI2/ST3 and IncI1 plasmids, as well as multiple resistance genes across different antibiotic classes, including *bla*_NDM-5_, consistent with a multidrug-resistant profile (Table 1). The *bla*_NDM-5_ gene was located on the IncHI2/ST3 plasmid which was over 260 kb in size with a GC content of 46.3%, and also carried 14 other antimicrobial resistance genes (Table 1). GD24LH266S shared an almost identical resistance gene and plasmid profile, except for the absence of *bla*_NDM-5_ and IncI1 plasmid (Table 1). To our knowledge, this represents a rare observation of *bla*_NDM-5_ in *S*. Stanley from a pediatric diarrhea case, highlighting the public health concern posed by foodborne *S*. Stanley carrying this resistance gene.

**TABLE 1:** Characterization of *Salmonella* isolates carried by the infant patient^*a*^

Isolate	MLST	Serotype	Plasmid type (pDLST)	Resistance genes	Antimicrobial profile (MIC, μg/mL)	Antimicrobial (KB, mm)
GD24LH212S	ST29	Stanley	**IncHI2/HI2A(ST3)**, IncI1(α)	***ARR-3***, ***aac(3)-IVa***, *aac(6′)-Iaa*, ***aadA2***, ***aph(4)-Ia**, **bla***_**NDM-5**_, ***bla***_**OXA-10**_, ***bla***_**TEM-1B**_, ***cmlA1***,***dfrA14***, ***floR***, ***qnrS1***, ***sul3***, ***tet*****(A)**	Resistant: CAZ (≥64), CRO (≥64), CSL (≥64), FEP (16), AMC (≥32), TZP (≥128), LVX (2), SXT (≥16/304), ETP (≥64), IPM (≥64) Susceptible: TGC (1)	Resistant: PEN (6), CZO (6) Intermediate: CIP (25) Susceptible: AZM (23)
GD24LH266S	ST29	Stanley	**IncHI2/HI2A(ST3)**	***ARR-3***, ***aac(3)-IVa***, *aac(6′)-Iaa*, ***aadA2***, ***aph(4)-Ia***, ***bla***_**OXA-10**_, ***bla***_**TEM-1B**_, ***cmlA1***, ***dfrA14***,***floR***, ***qnrS1***, ***sul3***, ***tet*****(A)**	Resistant: SXT (≥16/304) Intermediate: LVX (1) Susceptible: CAZ (0.25), CRO (≤0.25), CSL (≤8), FEP (≤0.12), AMC (4), TZP (≤4), ETP (≤0.12), IPM (≤0.25)	Resistant: PEN (6) Susceptible: CZO (32), CIP (25), AZM (23)

^*a*^ Bold: bla_NDM-5_-positive plasmids and their resistance genes. MIC, minimal inhibitory concentration; CAZ, ceftazidime; CRO, ceftriaxone; CSL, cefoperazone-sulbactam; FEP, cefepime; AMC, amoxicillin-clavulanate; TZP, piperacillin-tazobactam; LVX, levofloxacin; TGC, tigecycline; SXT, trimethoprim-sulfamethoxazole; ETP, ertapenem; IPM, imipenem; PEN, penicillin; CZO, cefazolin; ATM, aztreonam; CIP, ciprofloxacin; AZM, azithromycin.

**Fig 1:**
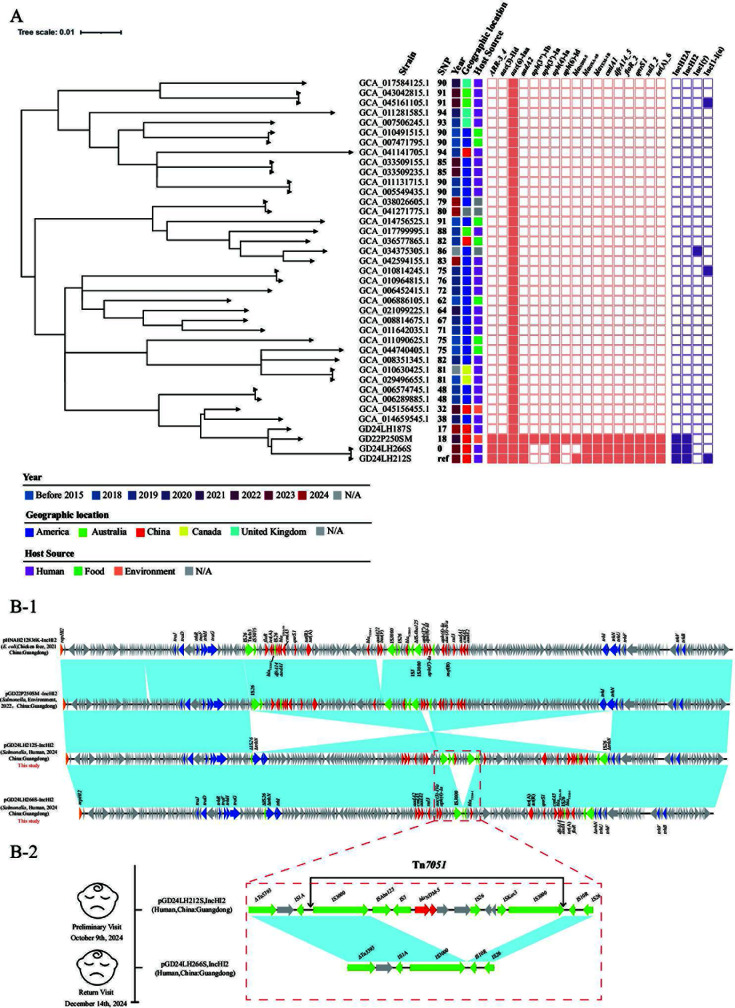
(**A**) Phylogenetic tree and heatmap of *Salmonella* ST29 isolates. (**B-1**) Comparison of *bla*_NDM-5_-IncHI2/ST3 plasmids with similar plasmids. (**B-2**) Schematic illustration of isolates from the pediatric case at initial (GD24LH212S) and follow-up (GD24LH266S) visits. Orange, red, green, and blue arrows represent plasmid replication genes, antimicrobial resistance genes, mobile element genes, and conjugative transfer genes, respectively. Light blue shading denotes regions of shared 99% homology among the different plasmids.

Phylogenetic analysis of core genomes, using GD24LH212S as the reference, revealed that GD24LH266S shared an identical core genome (0 SNP differences) and clustered closely with global ST29 *S*. Stanley strains (17–94 SNP differences relative to the reference) publicly available sequences in GenBank as well as additional *Salmonella* genomes sequenced in our laboratory, including isolates from human, food, and environment across China and other regions (Fig. 1A). Notably, GD22P250SM (GCA_037164745.1, Guangzhou, 2022), an ST29 *S*. Stanley strain from a swine slaughterhouse in our previous study, carried a *bla*_NDM-5_-bearing IncHI2/ST3 plasmid (9) and differed from GD24LH212S by only 18 SNPs. Similarly, GD24LH187S (Shenzhen, 2024, laboratory collection), obtained from the feces of a healthy individual, differed by 17 SNPs but lacked the *bla*_NDM-5_-bearing plasmid. Taken together, these findings indicate the potential for cross-host transmission and community spread of NDM-producing ST29 *S*. Stanley in Guangdong province of China.

A linear comparison of pHNGD24LH212S from GD24LH212S and pHNGD24LH266S from GD24LH266S with IncHI2/ST3 plasmids deposited in GenBank and plasmids obtained in our lab revealed that pHNGD24LH212S and pHNGD24LH266S were highly similar to pHNAH212836K (CP104628.1, China, chicken feces, 2021) (10) and pHNGD22P250SM (GCA_037164745.1, China, environmental sample, 2022) (9), with an average sequence identity of 99% and coverage of 98% (Fig. 1B-1). In contrast, the variable regions of pHNGD24LH212S and pHNGD24LH266S exhibited inversions compared with pHN22P250SM, likely driven by the insertion and transposition of IS elements such as IS*26*. Conjugation was performed with GD24LH212S (donor) and *Escherichia coli* J53 (recipient) at 25°C and 30°C. Donor and recipient cells were co-incubated in antibiotic-free broth for 10 h, and mixtures were plated onto meropenem and sodium azide supplemented selective agar. No transconjugants were recovered under either condition. Conjugation assays further demonstrated that both plasmids had lost transferability, which may be attributable to IS*26*-mediated truncation of *trhN* (Fig. 1B-1).pHNGD24LH212S and pHNGD24LH266S are highly similar, with differences only observed in the variable regions. pHNGD24LH266S, compared to pHNGD24LH212S, lacks Tn*7051* (IS*3000*-ΔIS*Aba125*-IS*5-bla*_NDM-5_-*ble*_MBL_-*trpF-dsbC*-IS*26-umuD*-∆IS*Kox3*-IS*3000*), and this observation may be associated with recombination of the two copies of IS*3000* in the same orientation, possibly leading to the insertion or loss of resistance modules (Fig. 1B-2). Competitive assays further showed that GD24LH212S carrying Tn*7051* had only a slight fitness advantage over GD24LH266S under antibiotic-free conditions (Fig. S1). This finding, together with the absence of antimicrobial treatment before 14 December, suggests that Tn*7051* loss may have been facilitated by selective pressure.

Previous epidemiological surveillances identified *S*. Stanley as a persistent pathogen disproportionately affecting pediatric (0–5 years) and geriatric (>60 years) populations, with recurrent isolation from commercial aquatic products and food matrices across multiple regions (11–13). Our previous detection of NDM-5-producing *S*. Stanley in slaughterhouse environments points to potential transmission nodes within the food chain. Phylogenetic analysis further demonstrates that this pathogen can undergo cross-host transmission through food chain pathways, facilitating its spread in human populations. Of particular concern, this case was epidemiologically linked to inadequate domestic food handling practices, specifically delayed cleaning of feeding bottles, establishing a direct connection between hygiene behaviors and clinical outcomes. In addition, our findings show that *S*. Stanley ST29 can persist in the infant gut for over 2 months, causing prolonged diarrhea likely due to the lack of timely diagnosis and targeted antimicrobial therapy. Interestingly, in the absence of antibiotic pressure, the *bla*_NDM-5_-carrying plasmid underwent Tn*7051*-mediated loss. Competition assays indicated that the Tn*7051-bla*_NDM-5_ module imposed only a minor fitness cost on the host strain, consistent with previous reports (14). This suggests that other factors may also drive the instability of *bla*_NDM-5_, which warrants further investigation. As carbapenem-resistant *Salmonella* represents a particular threat to infant health, improved household hygiene is vital to prevent foodborne infections in this population.

In summary, this study documents the first isolation of *bla*_NDM-5_-IncHI2/ST3 plasmid-harboring *S*. Stanley ST29 strain from an infant diarrheal case. Our findings revealed two urgent threats: mobile genetic elements spreading resistance genes through bacterial populations, while weaknesses in food safety systems allow contaminated food to reach vulnerable groups. They underscore the need for integrated One-Health surveillance and targeted interventions, particularly food-handler education, to disrupt transmission of high-risk-resistant clones.

## Data Availability

The complete genome sequences of GD24LH212S and GD24LH266S have been deposited in GenBank under accession numbers JBRTYF000000000.1 and JBRTYE000000000.1, respectively.

## SUPPLEMENTAL MATERIAL

Fig. S1 aac.01471-25-s0001.pdf Relative fitness of GD24LH212S and GD24LH266S in competition assays.

ASM does not own the copyrights to Supplemental Material that may be linked to, or accessed through, an article. The authors have granted ASM a non-exclusive, world-wide license to publish the Supplemental Material files. Please contact the corresponding author directly for reuse.
