# Supplementary material for: Salmonella Stanley ST29 carrying IncHI2/ST3-blaNDM-5 plasmid emerged in a 4-month-old infant with diarrhea
Source: Antimicrob Agents Chemother. 2026 Feb 4;70(3):e01471-25. doi: 10.1128/aac.01471-25 (PMC12959088; doi:10.1128/aac.01471-25)
Supplement: Fig. S1 — Relative fitness of GD24LH212S and GD24LH266S in competition assays. [file aac.01471-25-s0001.pdf]

Supplemental Material

Figure S1 Relative fitness of GD24LH212S and GD24LH266S in competition assays

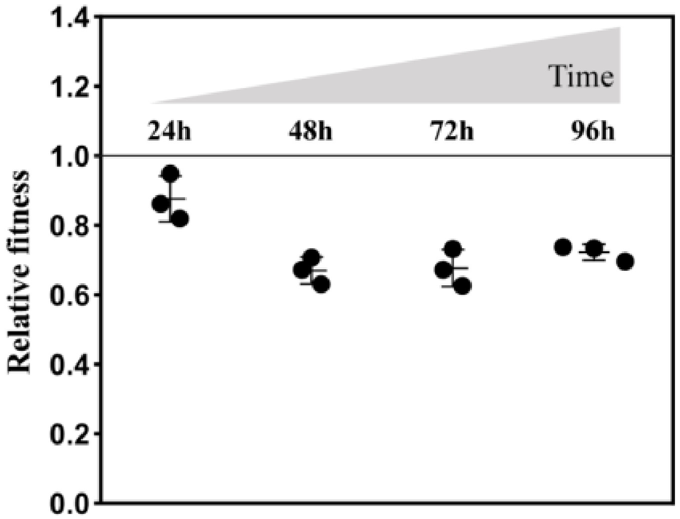

The relative fitness (RF) was calculated using the following formula: $RF=(\log_{10} S_{2dt}-\log_{10} S_{2d0})/(\log_{10} S_{1dt}-\log_{10} S_{1d0})$, where RF represents the relative fitness of strain S2 compared to strain S1.Here $S1_{d0}$ and $S1_{dt}$ denote the cell densities (CFU/mL) of strain S1 at the start and end of the competition, respectively, while $S2_{d0}$ and $S2_{dt}$ represent the corresponding values for strain S2. Each competition experiment was performed with three biological replicates.
